# Alcohol consumption, physical activity, and chronic disease risk factors: a population-based cross-sectional survey

**DOI:** 10.1186/1471-2458-6-118

**Published:** 2006-05-03

**Authors:** Kenneth J Mukamal, Eric L Ding, Luc Djoussé

**Affiliations:** 1 Division of General Medicine and Primary Care, Beth Israel Deaconess Medical Center and Harvard Medical School, Boston, Massachusetts, US; 2 Departments of Epidemiology and Nutrition, Harvard School of Public Health, Boston, Massachusetts, US; 3 Division of Aging, Brigham and Women's Hospital and Harvard Medical School, Boston, Massachusetts, US

## Abstract

**Background:**

Whether the association of alcohol consumption and cardiovascular disease is the product of confounding and the degree to which this concern applies to other behaviors are unclear.

**Methods:**

Using the 2003 Behavioral Risk Factor Surveillance System, a population-based telephone survey of adults in the US, we compared chronic disease risk factors between 123,359 abstainers and 126,674 moderate drinkers, defined as intake of ≤ 2 drinks per day among men and ≤ 1 drink per day among women, using age- and sex- and multivariable-adjusted models. We also compared sedentary and active individuals, defined as moderate physical activity ≥ 30 minutes per day for ≥ 5 days per week, or vigorous activity for ≥ 20 minutes per day on ≥ 3 days.

**Results:**

Chronic disease risk factors and features of unhealthy lifestyle were generally more prevalent among abstainers than drinkers in age- and sex-adjusted analyses, but these differences were generally attenuated or eliminated by additional adjustment for race and education. For low fruit and vegetable intake, divorced marital status, and absence of a personal physician, adjustment for race and education reversed initially positive age- and sex-adjusted associations with abstention. Comparison of sedentary and active individuals produced similar findings, with generally lower levels of risk factors among more physical active individuals.

**Conclusion:**

The differences between abstainers and drinkers are attenuated after adjustment for limited sociodemographic features, and sedentary and active individuals share a similar pattern. Although observational studies of both factors may be susceptible to uncontrolled confounding, our results provide no evidence that moderate drinking is unique in this regard. Ultimately, randomized trials of all such lifestyle factors will be needed to answer these questions definitively.

## Background

Moderate alcohol consumption, typically defined as up to 2 drinks per day for men and 1 drink per day for women, has been consistently associated with lower risk of coronary heart disease in observational studies. At least two meta-analyses have come to consistent conclusions about the magnitude of this association [1,2], and it is further supported by the established effects of moderate drinking on high-density lipoprotein cholesterol and other cardiovascular risk factors [3,4].

For a variety of reasons, the observed inverse association between moderate drinking and risk of coronary heart disease remains controversial. Most importantly, no long-term randomized clinical trial of alcohol consumption has been conducted. Although observational evidence and randomized trials generally yield similar findings [5], recent examples suggest that, at a minimum, the two types of evidence can sometimes be difficult to reconcile [6]. For moderate drinking, several authors have raised particular concern about the possibility of uncontrolled or residual confounding, in which unmeasured or poorly measured factors that differ between drinkers and abstainers are responsible for the apparently lower risk among drinkers [7-10]. In an analysis of the Behavioral Risk Factor Surveillance System (BRFSS) [9], Naimi and colleagues provided a particularly vivid example of this concern. The authors examined 30 factors potentially associated with poor health to varying degrees. Of these, 90% were more common among abstainers than moderate drinkers in age- and sex-adjusted analyses. This analysis has spurred widespread discussion about the limits of observed studies of moderate drinking [11].

Unfortunately, moderate drinking is not the only lifestyle factor associated with lower risk of incident myocardial infarction that has not been formally tested in a long-term randomized controlled trial of clinical events. For example, although physical activity is widely recommended for prevention of cardiovascular disease [12], this recommendation relies explicitly on observational evidence bolstered by "biological plausibility" [12]. Despite this fact, concern about the possibility of confounding in studies of moderate drinking has overshadowed similar concerns regarding physical activity.

To evaluate the association of moderate drinking with lifestyle and personal characteristics that could confound its association with coronary heart disease, we undertook a reanalysis of the 2003 BRFSS, with two aims. First, we sought to determine whether moderate drinking was consistently associated with a lower-risk profile after adjustment for basic demographic features, as studies of alcohol and coronary heart disease have generally adjusted for at least a few such potential confounders. Second, we compared the risk profiles of physical activity and moderate drinking, in an effort to determine whether moderate drinking is disproportionately susceptible to potential confounding.

## Methods

### Survey design

The BRFSS, administered by the Centers for Disease Control and Prevention, is an ongoing telephone-based data collection program designed to collect uniform, state-specific data on preventive health practices and risk behaviors that are linked to chronic diseases, injuries, and preventable infectious diseases in the adult population (18 years of age or older) living in households in the 50 states, the District of Columbia, Puerto Rico, Guam, and the Virgin Islands [13]. Factors assessed by the BRFSS include tobacco use, health care coverage, HIV/AIDS knowledge and prevention, physical activity, and fruit and vegetable consumption. Data are collected from a random sample of adults (one per household) through a yearly telephone survey conducted by state health personnel or contractors; overall, about 95% of US households have telephones.

The questionnaire has three parts: 1) the core component; 2) optional modules; and 3) state-added questions. The 2003 core and module questionnaires are publicly accessible [14]. The core component is a standard set of questions asked by all states. It includes queries about current health-related perceptions, conditions, and behaviors, as well as demographic questions. The optional modules are sets of questions on specific topics that states elect to use on their questionnaires.

In the BRFSS, sampled telephone numbers represents a probability sample of all households with telephones in a given state. All US states used a disproportionate stratified sample (DSS) design. Puerto Rico, Guam, and the U.S. Virgin Islands used a simple random sample design. In the type of DSS design most commonly used in the BRFSS, telephone numbers are divided into high-density and medium-density strata based upon the proportion of numbers expected to belong to households. The two strata are sampled separately to obtain a probability sample of all households with telephones.

In 2003, all states and territories used computer-assisted telephone interviewing. Following guidelines provided by CDC, state health personnel or contractors conduct interviews. The core portion of the questionnaire lasts an average of 10 minutes. All interviewers are given specific training on the BRFSS questionnaire and procedures. At least fifteen call attempts are made to each unanswered telephone number.

In 2003, a total of 264,684 individuals (104,400 men and 160,284 women) participated. The median cooperation rate, defined as the proportion of all respondents interviewed among all eligible units that were actually contacted, was 74.8% and ranged from 60.1% in California to 91.9% in Puerto Rico.

To ensure representativeness to the target population, probability sampling and post-stratification weights are used. Such poststratification serves as a blanket adjustment for both noncoverage and nonresponse and forces the total number of cases to equal population estimates for each geographic stratum.

The BRFSS informs all respondents at the outset that the survey is anonymous and confidential, that it collects no personally identifying information, and that answering any or all questions is entirely voluntary; consent is presumed on the basis of willingness to participate. The protocol for our analyses was subjected to ethics review by the Beth Israel Deaconess Medical Center Committee on Clinical Investigations (protocol 2005P-000328), which provided an exemption from continuing review.

### Assessment of alcohol and physical activity

Participants reported the number of days that they consumed at least one drink in the previous 30 days and the average number of drinks that they consumed on those days. A drink was defined as "1 can or bottle of beer, 1 glass of wine, 1 can or bottle of wine cooler, 1 cocktail, or 1 shot of liquor." Drinking frequency and quantity consumed per drinking day were multiplied to yield the BRFSS measure of total alcohol consumption. As in previous analyses from other investigators [9], we compared abstainers to moderate drinkers, defined as men who consumed 2 drinks per day or less and women who consumed 1 drink per day or less; heavier drinking participants were not included. Abstainers were defined as individuals who reported no alcohol consumption in the previous 30 days.

In 2003, the BRFSS included a core module on physical activity. Participants separately reported their level of moderate activity, defined as causing a small increase in breathing or heart rate with examples of brisk walking, bicycling, vacuuming, and gardening, and vigorous activity, defined as causing a large increase in breathing or heart rate with examples of running, aerobics, and heavy yard work. For each type, participants reported whether they engaged in such activity for at least 10 minutes in a typical week and if so, the number of days per week they did so and the total time spent each day. The BRFSS established a physical activity goal of moderate physical activity 30 or more minutes per day for 5 or more days per week, or vigorous activity for 20 or more minutes per day on 3 or more days per week. We compared active individuals who met this goal with sedentary individuals who did not.

### Other behavioral characteristics

We adopted a similar approach to previous authors [9], examining a full series of potential risk factors, whether or not they were known to be directly related to coronary heart disease. We used four categories for marital status (married, divorced, widowed, and never-married), three for income (<$25,000, $25,000–$49,000, or $50,000 or more per year), and five for self-reported health (excellent, very good, good, fair, and poor). Leisure-time physical activity was defined as any leisure time physical activity or exercise during the past 30 days other than one's regular job. Adequate intake of fruits and vegetables required intake of 5 or more servings per day. Lack of influenza vaccination within the past year was considered a risk factor among participants aged 65 years and older. Receipt of colonoscopy or sigmoidoscopy included any such procedure performed within the prior 10 years among individuals 50 years and older, and cholesterol screening included any screening within the last 5 years. Participants self-reported the presence of physician-diagnosed medical illnesses including diabetes, hypertension, hypercholesterolemia, and arthritis. Participants also separately reported the number of days within the last 30 that their physical health or mental health was "not good" and the number of days that poor physical or mental health interrupted their usual activities. In ten states, respondents reported loss of any permanent teeth because of tooth decay or gum disease, excluding teeth lost because of injury or orthodontics.

We also included the HIV/AIDS risk factor, as this was not included in previous work [9] but is an established BRFSS risk factor among adults less than 65 years of age. This risk factor included any of the following activities within the past year: use of intravenous drugs, treatment for a sexually transmitted disease, payment or receipt of money or drugs in exchange for sex, or anal sex without a condom.

### Statistical analyses

For univariate and bivariate comparisons, we present prevalence estimates weighted to the underlying population distribution. For maximum comparability with previous work [9], we performed multivariable analyses using logistic regression, with a dependent variable of abstention (versus moderate drinking, with heavier drinking excluded) or sedentary lifestyle (versus physically active). In such cases, we present weighted odds ratios with their 95% confidence intervals. Each behavioral factor was examined as an independent variable or a series of independent indicator variables when multiple categories were defined. We present both age- and sex-adjusted analyses and analyses additionally adjusted for education and race, two readily-measured variables that are commonly adjusted for (or stratified by) in many epidemiological studies. In these additionally-adjusted analyses, race was categorized into six groups (non-Hispanic white, non-Hispanic black, Hispanic, Asian, American Indian/Alaskan native, and other) and education into four groups (less than high school, high school, some college, and college graduate). Analyses shown include age as a continuous variable; alternate analyses that adjusted for age as a fractional polynomial [15] yielded qualitatively similar results. Finally, to estimate weighted prevalence ratios, we used Poisson regression [16]. We used Intercooled STATA 8.2 for Windows (StataCorp; College Station, TX; 2005) in all analyses to account for the sampling weights.

## Results

### Moderate drinking and behavioral characteristics

Of the 264,684 participating adults, 123,359 reported abstention and 126,674 reported moderate drinking. Likewise, 114,287 adults met the BRFSS criteria for adequate physical activity, while 134,630 were sedentary. Moderate drinking and physical activity were strongly associated, with 50% of moderate drinkers but 60% of abstainers reporting sedentary levels of activity (p < 0.001).

Table 1 shows the relationship of the prevalence of various risk factors with moderate drinking or abstention after adjustment for age and sex and after further adjustment for race and education. In initial analyses, most risk factors were positively associated with a higher prevalence of abstention, although cigarette smoking and behaviors linked to HIV risk were associated with a lower prevalence. Further adjustment for education and race attenuated all of the associations of risk factors with a higher prevalence of abstention. For unmarried marital status, employment, and hypercholesterolemia, the multivariable-adjusted associations were null. Most of the other associations were modest in magnitude, with the exceptions of income, leisure-time physical activity, obesity, medical equipment use, and self-reported health status.

Further adjustment for race and education also had two other notable effects. First, it magnified the inverse associations of abstention with cigarette smoking and HIV risk seen in age- and sex-adjusted analyses. Second, it changed the direction of the associations of abstention with low fruit and vegetable intake, divorced marital status, and the absence of a personal physician. In all three cases, abstention was positively related to these risk factors in age- and sex-adjusted analyses, but was inversely associated after further adjustment. Among current smokers, abstention was also related to a lower adjusted likelihood of reporting daily smoking (odds ratio 0.75; 95% confidence interval, 0.69–0.82).

### Physical activity and behavioral characteristics

The relationships of physical activity with behavioral characteristics strongly paralleled those of moderate drinking (Table 2). With the exception of income, the magnitude of the associations of individual risk factors with physical activity and alcohol intake were generally comparable. Low physical activity was positively associated with nearly all other risk factors; unmarried marital status, HIV risk, and former smoking were the only factors that were inversely associated. Of particular note, current cigarette smoking and low intake of fruits and vegetables, which were inversely associated with abstention from alcohol, were positively associated with low physical activity.

We present odds ratios for maximal comparability with previous studies. However, both abstention and sedentary lifestyle are very common. As a result, all of the odds ratios reported here (and in previous work [9]) overestimate prevalence ratios, which are also shown in Tables 1 and 2. As particularly noteworthy examples, the adjusted odds ratios for abstention associated with poor physical health and poor self-reported health status were 1.79 (95% confidence interval, 1.69–1.88) and 3.08 (95% confidence interval, 2.81–3.37), respectively. In contrast, the corresponding adjusted prevalence ratios using Poisson regression were 1.26 (95% confidence interval, 1.23–1.28) and 1.50 (95% confidence interval, 1.45–1.54), which imply associations of considerably more modest magnitude.

## Discussion

In this population-based cross-sectional study, levels of cardiovascular risk factors and features of unhealthy lifestyle were generally more common among abstainers than drinkers in age- and sex-adjusted analyses, but these differences were attenuated, eliminated, or reversed by additional adjustment for race and education alone. A similar pattern was evident among sedentary and physically active individuals.

This study cannot determine whether the observed relationships of alcohol consumption or physical activity with risk of coronary heart disease are confounded. Such a determination requires knowledge about the independent relationships of potential confounders with both exposure and outcome, the magnitude of such relationships, the nature of plausible causal pathways, and the degree to which potential confounders are appropriately measured [17,18]. As a result, even if moderate drinking or physical activity were independently associated with every potential risk factor, it cannot be directly assumed that these risk factors explain the observed relationships of alcohol consumption or physical activity with cardiovascular risk. This has already been described as a limitation of a previous analysis of the association of moderate drinking with behavioral characteristics [19].

The similarity in our findings regarding alcohol consumption and physical activity warrants careful examination. Our results do not imply that neither of these factors is causally related to lower risk of coronary heart disease, nor do they exclude the possibility that only one factor is causally related. However, our results do suggest that concerns about confounding should be applied generally in observational research, and not limited to specific exposures. Given the similarities in existing evidence about moderate drinking and physical activity, despite widespread consensus that physical activity prevents cardiovascular disease, there seems no reason to assume that studies on moderate alcohol consumption are uniquely confounded.

We examined risk factors of several types. Some of these, such as measures of socioeconomic status or general health, are apt to be true confounders; that is, they appear to influence both exposure (whether moderate drinking or physical activity) and outcome (coronary heart disease). Others, such as colorectal cancer screening, are not plausibly associated with either exposure or outcome. Finally, some differences between drinkers and abstainers, or between sedentary and active adults, may be caused by alcohol or activity per se and hence should best be considered intermediates. For example, randomized clinical studies demonstrate that moderate alcohol intake directly improves insulin sensitivity [20,21], perhaps by raising adiponectin levels [21,22], and hence differences in diabetes rates may be related to biological effects of alcohol consumption rather than confounding. The same may be true for the lower prevalence of hypertension among moderate drinkers [23,24]. Straightforward counting of risk factors tends to blur these key distinctions.

Adjustment for race and education influenced the relationships of moderate drinking with putative risk factors in a number of ways. In most cases, it attenuated the observed relationships, but some relationships, such as those with income and leisure-time physical activity, remained quite strong, while others, such as unemployment and hypercholesterolemia, became null. In this regard, our findings suggest that particular importance be paid to careful measurement of income and activity in studies of the health effects of alcohol intake. In still other cases, multivariable adjustment revealed otherwise-obscured associations of abstention with lower levels of risk factors, such as with divorce, low fruit and vegetable intake, and not having a personal doctor. Lastly, it magnified the strong relationships of moderate drinking with both prevalence and intensity of cigarette smoking, which are among the most potent risk factors for cardiovascular disease of the other characteristics included here. In sum, we would caution against simple age- and sex-adjusted analyses in studies of the association of moderate drinking with either risk of cardiovascular disease or potential risk factors.

The BRFSS does not contain questions about former drinking, a longstanding concern in studies of health effects of alcohol [25]. As a result, the pool of abstainers may contain individuals who quit drinking, in some cases because of health problems. For this reason, many studies have used occasional drinkers, rather than abstainers, as the reference category [26,27], or have separated former drinkers from longer-term abstainers [27,28]. Both of these approaches are preferable to unrestricted comparison of all drinkers with all teetotalers, which tends to exaggerate the cross-sectional association of abstention with markers of poor health. The same concern is also apt to be true for physical activity.

Other limitations of our study also warrant discussion. All of the information in the BRFSS is self-reported, and no independent validation of reported alcohol intake or physical activity can be made, although there is no reason to believe that these variables are measured with less accuracy in BRFSS than in other studies. The BRFSS is also a cross-sectional survey, and many of the associations evaluated here might differ in prospective analyses.

## Conclusion

In summary, both moderate drinking and physical activity are associated with healthier lifestyle characteristics after adjustment for age and sex, although these associations are attenuated after modest multivariable adjustment for race and education alone and their absolute magnitude is modest. Neither moderate drinking nor physical activity have been proven to prevent cardiovascular disease in randomized trials, and hence observational studies of both factors may be susceptible to uncontrolled confounding. Nonetheless, our results provide no evidence that moderate drinking is unique in this regard, at least when compared to physical activity, which is widely assumed to prevent cardiovascular disease. Ultimately, randomized trials of all putative lifestyle factors, including multiple aspects of diet and weight loss, will be needed to answer these questions definitively.

## Abbreviations

BRFSS – Behavioral Risk Factor Surveillance System

## Competing interests

The author(s) declare that they have no competing interests.

## Authors' contributions

KJM performed the analyses and wrote the manuscript. ELD and LD participated in the conception and design of the study, made substantive recommendations to the analyses and their interpretation, and critically revised the manuscript.

## Pre-publication history

The pre-publication history for this paper can be accessed here:



## Figures and Tables

**Table 1:** Weighted prevalence and odds and prevalence ratios (and 95% confidence intervals) for abstention according to behavioural risk factors among respondents to the 2003 Behavioral Risk Factor Surveillance System survey.

	Abstainers	Odds Ratio		Prevalence Ratio
	
	N (%)	Age-Sex	+Race-Educ	Age-Sex-Race-Educ
***DEMOGRAPHICS***				
**Marital Status**				
Married	68236 (45)	1.0	1.0	1.0
Divorced	19858 (48)	1.04 (1.00–1.09)	0.93 (0.89–0.97)	0.97 (0.95–0.99)
Widowed	18940 (66)	1.44 (1.36–1.53)	1.16 (1.09–1.24)	1.02 (1.00–1.04)
Unmarried	15912 (43)	1.16 (1.10–1.21)	1.04 (0.98–1.09)	1.02 (0.99–1.04)
**Income ($/yr)**				
≥50,000	25868 (32)	1.0	1.0	1.0
25,000–49,999	33572 (47)	1.81 (1.74–1.89)	1.53 (1.46–1.59)	1.29 (1.26–1.33)
<25,000	43693 (60)	2.95 (2.83–3.08)	2.06 (1.97–2.17)	1.46 (1.42–1.50)
**Employed**				
Yes	117025 (46)	1.0	1.0	1.0
No	5947 (49)	1.28 (1.19–1.39)	1.06 (0.98–1.15)	1.04 (1.00–1.07)
***BEHAVIORAL FACTORS***				
**Smoking Status**				
Never	70729 (50)	1.0	1.0	1.0
Former	29980 (43)	0.68 (0.65–0.70)	0.66 (0.64–0.69)	0.83 (0.81–0.85)
Current	22307 (41)	0.75 (0.72–0.78)	0.60 (0.58–0.63)	0.79 (0.77–0.81)
**Leisure-Time Physical Activity**				
Yes	82794 (42)	1.0	1.0	1.0
No	40348 (61)	2.02 (1.95–2.10)	1.62 (1.56–1.68)	1.23 (1.21–1.25)
**5+ Servings of Fruits/Vegetables**				
Yes	29990 (49)	1.0	1.0	1.0
No	92003 (46)	0.99 (0.95–1.03)	0.90 (0.86–0.93)	0.95 (0.93–0.96)
**HIV Risk**				
Low	83159 (44)	1.0	1.0	1.0
High	2080 (34)	0.70 (0.63–0.78)	0.59 (0.53–0.66)	0.74 (0.69–0.79)
***ACCESS TO CARE***				
**Health Insurance**				
Yes	106124 (46)	1.0	1.0	1.0
No	16791 (51)	1.50 (1.43–1.58)	1.10 (1.05–1.16)	1.05 (1.03–1.08)
**Personal Doctor**				
Yes	103825 (48)	1.0	1.0	1.0
No	18073 (43)	1.07 (1.03–1.12)	0.90 (0.86–0.94)	0.95 (0.92–0.97)
**Could Afford to See MD**				
Yes	106128 (46)	1.0	1.0	1.0
No	16891 (52)	1.34 (1.27–1.40)	1.08 (1.03–1.14)	1.04 (1.02–1.06)
**Influenza Vaccination**				
Yes	23047 (58)	1.0	1.0	1.0
No	10980 (65)	1.36 (1.27–1.46)	1.17 (1.08–1.26)	1.05 (1.03–1.08)
**Had Cholesterol Checked**				
Yes	93639 (47)	1.0	1.0	1.0
No	25290 (46)	1.25 (1.20–1.30)	1.06 (1.01–1.10)	1.03 (1.01–1.05)
**Sigmoidoscopy or Colonoscopy**				
Yes	5308 (53)	1.0	1.0	1.0
No	6175 (56)	1.23 (1.12–1.36)	1.10 (1.00–1.22)	1.04 (1.00–1.08)
***MEDICAL HISTORY***				
**Diabetes**				
No	107894 (45)	1.0	1.0	1.0
Yes	15289 (68)	2.35 (2.22–2.49)	2.07 (1.95–2.20)	1.31 (1.28–1.33)
**Hypertension**				
No	80231 (43)	1.0	1.0	1.0
Yes	42855 (56)	1.43 (1.38–1.48)	1.29 (1.25–1.34)	1.12 (1.10–1.14)
**Hypercholesterolemia**				
No	61238 (45)	1.0	1.0	1.0
Yes	37540 (49)	1.04 (1.00–1.08)	1.02 (0.98–1.05)	1.01 (0.99–1.03)
**Arthritis**				
No	75627 (44)	1.0	1.0	1.0
Yes	45140 (54)	1.22 (1.18–1.26)	1.17 (1.13–1.22)	1.07 (1.06–1.09)
**Uses Medical Equipment**				
No	107770 (45)	1.0	1.0	1.0
Yes	13023 (68)	2.11 (1.98–2.25)	1.89 (1.76–2.02)	1.25 (1.22–1.27)
**Any Teeth Removed**				
No	8228 (37)	1.0	1.0	1.0
Yes	11271 (51)	1.56 (1.45–1.68)	1.22 (1.13–1.32)	1.12 (1.07–1.16)
**Body-Mass Index (kg/m**^**2**^**)**				
<18	1757 (56)	1.64 (1.40–1.92)	1.45 (1.23–1.70)	1.19 (1.11–1.27)
18.0–24.9	41756 (43)	1.0	1.0	1.0
25.0–29.9	40095 (44)	1.11 (1.07–1.15)	1.07 (1.03–1.12)	1.03 (1.01–1.06)
30.0–34.9	19787 (51)	1.45 (1.38–1.52)	1.33 (1.26–1.39)	1.14 (1.12–1.17)
35.0–39.9	7568 (57)	1.82 (1.69–1.95)	1.65 (1.54–1.78)	1.25 (1.21–1.29)
≥40.0	4662 (63)	2.24 (2.03–2.46)	1.91 (1.73–2.12)	1.31 (1.26–1.36)
**Current Asthma**				
No	111519 (46)	1.0	1.0	1.0
Yes	11356 (52)	1.18 (1.12–1.25)	1.16 (1.09–1.23)	1.07 (1.04–1.10)
**General Health Status**				
Excellent	20053 (38)	1.0	1.0	1.0
Very Good	33681 (40)	1.06 (1.01–1.10)	1.00 (0.96–1.05)	1.01 (0.98–1.03)
Good	38403 (49)	1.53 (1.46–1.60)	1.24 (1.18–1.29)	1.13 (1.10–1.16)
Fair	20441 (63)	2.52 (2.38–2.68)	1.79 (1.68–1.90)	1.30 (1.27–1.34)
Poor	10303 (76)	4.41 (4.04–4.81)	3.08 (2.81–3.37)	1.50 (1.45–1.54)
**14 or more days in the last month of...**				
**Poor Physical Health**				
No	99599 (44)	1.0	1.0	1.0
Yes	20246 (65)	2.08 (1.97–2.18)	1.79 (1.69–1.88)	1.26 (1.23–1.28)
**Poor Mental Health**				
No	106355 (46)	1.0	1.0	1.0
Yes	14118 (52)	1.27 (1.21–1.34)	1.10 (1.04–1.16)	1.05 (1.02–1.07)
**Limitation of Activities**				
No	109034 (45)	1.0	1.0	1.0
Yes	12465 (65)	2.06 (1.93–2.20)	1.76 (1.64–1.88)	1.25 (1.22–1.28)

**Table 2:** Odds ratios and 95% confidence intervals for prevalence of sedentary lifestyle according to behavioural risk factors among respondents to the 2003 Behavioral Risk Factor Surveillance System survey.

	Sedentary	Odds Ratio		Prevalence Ratio
	
	N (%)	Age-Sex	+Race-Educ	Age-Sex-Race-Educ
***DEMOGRAPHICS***				
**Marital Status**				
Married	75176 (54)	1.0	1.0	1.0
Divorced	22911 (57)	1.12 (1.08–1.17)	1.06 (1.02–1.11)	1.03 (1.01–1.05)
Widowed	17671 (70)	1.38 (1.29–1.47)	1.23 (1.15–1.31)	1.05 (1.03–1.08)
Unmarried	18524 (47)	0.96 (0.92–1.01)	0.91 (0.87–0.95)	0.95 (0.93–0.97)
**Income ($/yr)**				
≥50,000	36770 (47)	1.0	1.0	1.0
25,000–49,999	37340 (54)	1.29 (1.24–1.34)	1.18 (1.13–1.23)	1.08 (1.06–1.11)
<25,000	41776 (61)	1.72 (1.65–1.80)	1.39 (1.33–1.46)	1.16 (1.13–1.18)
**Employed**				
Yes	128141 (54)	1.0	1.0	1.0
No	6208 (53)	1.10 (1.02–1.18)	0.97 (0.90–1.05)	0.99 (0.96–1.03)
***BEHAVIORAL FACTORS***				
**Smoking Status**				
Never	69307 (54)	1.0	1.0	1.0
Former	35651 (54)	0.90 (0.86–0.93)	0.92 (0.89–0.96)	0.97 (0.96–0.99)
Current	29328 (55)	1.11 (1.07–1.15)	1.07 (1.03–1.11)	1.04 (1.02–1.06)
**Leisure-Time Physical Activity**				
Yes	86130 (46)	1.0	1.0	1.0
No	48371 (79)	4.07 (3.91–4.25)	3.75 (3.59–3.91)	1.60 (1.58–1.62)
**5+ Servings of Fruits/Vegetables**				
Yes	25503 (44)	1.0	1.0	1.0
No	107472 (57)	1.88 (1.81–1.95)	1.84 (1.77–1.91)	1.33 (1.31–1.36)
**HIV Risk**				
Low	96261 (52)	1.0	1.0	1.0
High	3230 (48)	0.98 (0.89–1.08)	0.90 (0.81–0.99)	0.95 (0.90–1.00)
***ACCESS TO CARE***				
**Health Insurance**				
Yes	117042 (54)	1.0	1.0	1.0
No	17265 (56)	1.27 (1.21–1.34)	1.06 (1.01–1.11)	1.03 (1.01–1.05)
**Personal Doctor**				
Yes	111667 (54)	1.0	1.0	1.0
No	21230 (54)	1.21 (1.15–1.26)	1.08 (1.04–1.13)	1.04 (1.02–1.06)
**Could Afford to See MD**				
Yes	117210 (54)	1.0	1.0	1.0
No	17145 (57)	1.25 (1.19–1.31)	1.11 (1.06–1.17)	1.05 (1.03–1.07)
**Influenza Vaccination**				
Yes	22162 (63)	1.0	1.0	1.0
No	10125 (65)	1.18 (1.10–1.28)	1.08 (1.00–1.17)	1.02 (1.00–1.05)
**Had Cholesterol Checked**				
Yes	101385 (54)	1.0	1.0	1.0
No	28929 (54)	1.31 (1.26–1.36)	1.21 (1.16–1.25)	1.09 (1.07–1.11)
**Sigmoidoscopy or Colonoscopy**				
Yes	5722 (60)	1.0	1.0	1.0
No	6472 (63)	1.20 (1.09–1.32)	1.14 (1.03–1.26)	1.05 (1.01–1.09)
***MEDICAL HISTORY***				
**Diabetes**				
No	120719 (53)	1.0	1.0	1.0
Yes	13759 (67)	1.52 (1.43–1.61)	1.38 (1.30–1.47)	1.12 (1.10–1.14)
**Hypertension**				
No	90050 (51)	1.0	1.0	1.0
Yes	44319 (62)	1.25 (1.21–1.30)	1.19 (1.15–1.24)	1.07 (1.06–1.09)
**Hypercholesterolemia**				
No	66274 (51)	1.0	1.0	1.0
Yes	41022 (59)	1.21 (1.17–1.26)	1.21 (1.16–1.25)	1.09 (1.07–1.10)
**Arthritis**				
No	85957 (52)	1.0	1.0	1.0
Yes	46649 (60)	1.10 (1.06–1.14)	1.09 (1.06–1.13)	1.04 (1.02–1.05)
**Uses Medical Equipment**				
No	119666 (53)	1.0	1.0	1.0
Yes	13470 (76)	2.27 (2.12–2.43)	2.13 (1.99–2.29)	1.26 (1.24–1.29)
**Any Teeth Removed**				
No	10628 (47)	1.0	1.0	1.0
Yes	12149 (59)	1.31 (1.22–1.41)	1.13 (1.05–1.21)	1.06 (1.03–1.10)
**Body-Mass Index (kg/m**^**2**^**)**				
<18	1710 (56)	1.30 (1.10–1.52)	1.21 (1.02–1.42)	1.09 (1.02–1.17)
18.0–24.9	45241 (49)	1.0	1.0	1.0
25.0–29.9	45722 (53)	1.15 (1.11–1.19)	1.13 (1.09–1.17)	1.06 (1.04–1.08)
30.0–34.9	21907 (59)	1.46 (1.39–1.53)	1.40 (1.33–1.47)	1.16 (1.14–1.19)
35.0–39.9	8159 (64)	1.80 (1.67–1.94)	1.72 (1.59–1.85)	1.25 (1.22–1.29)
≥40.0	4945 (71)	2.42 (2.18–2.69)	2.24 (2.01–2.49)	1.36 (1.32–1.41)
**Current Asthma**				
No	122542 (54)	1.0	1.0	1.0
Yes	11569 (56)	1.09 (1.03–1.15)	1.09 (1.03–1.15)	1.04 (1.01–1.06)
**General Health Status**				
Excellent	20959 (41)	1.0	1.0	1.0
Very Good	40670 (50)	1.40 (1.34–1.46)	1.39 (1.33–1.45)	1.19 (1.17–1.22)
Good	42489 (59)	2.01 (1.92–2.09)	1.84 (1.76–1.93)	1.36 (1.33–1.39)
Fair	20174 (68)	2.76 (2.60–2.92)	2.37 (2.23–2.52)	1.47 (1.44–1.51)
Poor	9914 (78)	4.28 (3.90–4.71)	3.71 (3.37–4.08)	1.65 (1.60–1.70)
**14 or more days in the last month of...**				
**Poor Physical Health**				
No	111357 (52)	1.0	1.0	1.0
Yes	20246 (69)	1.80 (1.71–1.90)	1.68 (1.59–1.77)	1.21 (1.19–1.23)
**Poor Mental Health**				
No	116616 (53)	1.0	1.0	1.0
Yes	15438 (59)	1.29 (1.23–1.36)	1.22 (1.16–1.29)	1.09 (1.07–1.11)
**Limitation of Activities**				
No	120281 (53)	1.0	1.0	1.0
Yes	12773 (70)	1.95 (1.82–2.09)	1.80 (1.68–1.93)	1.23 (1.21–1.26)

## References

[B1] Maclure M (1993). Demonstration of deductive meta-analysis: ethanol intake and risk of myocardial infarction. Epidemiol Rev.

[B2] Corrao G, Rubbiati L, Bagnardi V, Zambon A, Poikolainen K (2000). Alcohol and coronary heart disease: a meta-analysis. Addiction.

[B3] Rimm EB, Williams P, Fosher K, Criqui M, Stampfer MJ (1999). Moderate alcohol intake and lower risk of coronary heart disease: meta-analysis of effects on lipids and haemostatic factors. BMJ.

[B4] Langer RD, Criqui MH, Reed DM (1992). Lipoproteins and blood pressure as biological pathways for effect of moderate alcohol consumption on coronary heart disease. Circulation.

[B5] Concato J, Shah N, Horwitz RI (2000). Randomized, controlled trials, observational studies, and the hierarchy of research designs. N Engl J Med.

[B6] Ioannidis JP, Haidich AB, Pappa M, Pantazis N, Kokori SI, Tektonidou MG, Contopoulos-Ioannidis DG, Lau J (2001). Comparison of evidence of treatment effects in randomized and nonrandomized studies. JAMA.

[B7] Goldberg IJ (2003). To drink or not to drink?. N Engl J Med.

[B8] Freiberg MS, Samet JH (2005). Alcohol and coronary heart disease: the answer awaits a randomized controlled trial. Circulation.

[B9] Naimi TS, Brown DW, Brewer RD, Giles WH, Mensah G, Serdula MK, Mokdad AH, Hungerford DW, Lando J, Naimi S, Stroup DF (2005). Cardiovascular risk factors and confounders among nondrinking and moderate-drinking U.S. adults. Am J Prev Med.

[B10] Lieber CS (2003). Alcohol and health: a drink a day won't keep the doctor away. Cleve Clin J Med.

[B11] Jackson R, Broad J, Connor J, Wells S (2005). Alcohol and ischaemic heart disease: probably no free lunch. Lancet.

[B12] Thompson PD, Buchner D, Pina IL, Balady GJ, Williams MA, Marcus BH, Berra K, Blair SN, Costa F, Franklin B, Fletcher GF, Gordon NF, Pate RR, Rodriguez BL, Yancey AK, Wenger NK (2003). Exercise and physical activity in the prevention and treatment of atherosclerotic cardiovascular disease: a statement from the Council on Clinical Cardiology (Subcommittee on Exercise, Rehabilitation, and Prevention) and the Council on Nutrition, Physical Activity, and Metabolism (Subcommittee on Physical Activity). Circulation.

[B13] Centers for Disease Control and Prevention (2003). Behavioral Risk Factor Surveillance System Survey Data.

[B14] (2003). BRFSS Questionnaires. http://www.cdc.gov/brfss/questionnaires/pdf-ques/2003brfss.pdf .

[B15] Greenland S (1995). Dose-response and trend analysis in epidemiology: alternatives to categorical analysis. Epidemiology.

[B16] Spiegelman D, Hertzmark E (2005). Easy SAS calculations for risk or prevalence ratios and differences. Am J Epidemiol.

[B17] Greenland S, Pearl J, Robins JM (1999). Causal diagrams for epidemiologic research. Epidemiology.

[B18] Greenland S, Robins JM (1985). Confounding and misclassification. Am J Epidemiol.

[B19] Ellison RC, Rothman KJ, Zhang Y, Djousse L (2005). Cardiovascular risk factors and confounders among nondrinking and moderate-drinking U.S. adults [letter]. Am J Prev Med.

[B20] Davies MJ, Baer DJ, Judd JT, Brown ED, Campbell WS, Taylor PR (2002). Effects of moderate alcohol intake on fasting insulin and glucose concentrations and insulin sensitivity in postmenopausal women: a randomized controlled trial. JAMA.

[B21] Sierksma A, Patel H, Ouchi N, Kihara S, Funahashi T, Heine RJ, Grobbee DE, Kluft C, Hendriks HF (2004). Effect of moderate alcohol consumption on adiponectin, tumor necrosis factor-alpha, and insulin sensitivity. Diabetes Care.

[B22] Pischon T, Girman CJ, Rifai N, Hotamisligil GS, Rimm EB (2005). Association between dietary factors and plasma adiponectin concentrations in men. Am J Clin Nutr.

[B23] Thadhani R, Camargo CA, Stampfer MJ, Curhan GC, Willett WC, Rimm EB (2002). Prospective study of moderate alcohol consumption and risk of hypertension in young women. Arch Intern Med.

[B24] Foppa M, Fuchs FD, Preissler L, Andrighetto A, Rosito GA, Duncan BB (2002). Red wine with the noon meal lowers post-meal blood pressure: a randomized trial in centrally obese, hypertensive patients. J Stud Alcohol.

[B25] Shaper AG, Wannamethee G, Walker M (1989). Alcohol and the U-shaped curve. Lancet.

[B26] Tolstrup JS, Jensen MK, Tjonneland A, Overvad K, Gronbaek M (2004). Drinking pattern and mortality in middle-aged men and women. Addiction.

[B27] Rimm EB, Giovannucci EL, Willett WC, Colditz GA, Ascherio A, Rosner B, Stampfer MJ (1991). Prospective study of alcohol consumption and risk of coronary disease in men. Lancet.

[B28] Klatsky AL, Armstrong MA, Friedman GD (1986). Relations of alcoholic beverage use to subsequent coronary artery disease hospitalization. Am J Cardiol.

